# Are Tanzanian health facilities ready to provide management of chronic respiratory diseases? An analysis of national survey for policy implications

**DOI:** 10.1371/journal.pone.0210350

**Published:** 2019-01-07

**Authors:** Festo K. Shayo, Deogratius Bintabara

**Affiliations:** 1 Department of Global Health Entrepreneurship, Division of Public Health, Graduate School of Tokyo Medical and Dental University, Tokyo, Japan; 2 Department of Internal Medicine, Muhimbili University of Health and Allied Sciences, Dar es Salaam, Tanzania; 3 Department of Public Health, College of Health Sciences, The University of Dodoma, Dodoma, Tanzania; Ministry of Health and Sports, MYANMAR

## Abstract

**Introduction:**

Chronic respiratory diseases in Tanzania are prevalent and a silent burden to the affected population, and healthcare system. We aimed to explore the availability of services and level of health facilities readiness to provide management of chronic respiratory diseases and its associated factors.

**Methods:**

The current study is a secondary analysis of the 2014–2015 Tanzania Service Provision Assessment Survey data. Facilities were considered to have a high readiness to provide management of chronic respiratory diseases if they scored at least half (≥50%) of the indicators listed in each of the three domains (staff training and guideline, equipment, and basic medicines) as identified by World Health Organization-Service Availability and Readiness Assessment manual. Descriptive, unadjusted and adjusted logistic regression analyses were performed. A *P* value < 0.05 was taken to indicate statistical significance.

**Results:**

Out of 723 facilities included in this analysis, approximately one-tenth had a high readiness to provide management of chronic respiratory diseases. Less than 10% of the facilities had at least one staff who received training for management of chronic respiratory diseases. In an adjusted model, privately owned facilities [AOR = 3.3; 95% CI, 1.5–7.5], hospitals [AOR = 11.6; 95% CI, 5.0–27.2], health centres [AOR = 5.0; 95% CI, 2.4–10.7], and performance of routine management meeting [AOR = 3.3; 95% CI, 1.4–7.8] were significantly associated with high readiness to provide management for chronic respiratory diseases.

**Conclusion:**

Majority of Tanzanian health facilities have low readiness to provide management for chronic respiratory diseases. There is a need for the Tanzanian government to increase the availability of diagnostic equipment, medication, and to provide refresher training specifically in the lower-level and public health facilities for better management of chronic respiratory diseases and other non-communicable diseases.

## Introduction

Chronic respiratory diseases (CRDs) are diseases of the airways and other structures of the lungs commonly include chronic obstructive pulmonary disease (COPD), bronchial asthma, respiratory allergies, and occupational lung diseases [1]. Globally in 2016 CRDs, in particular, COPD and Asthma affected 251 [2] and 339 [3] million people respectively. In the same year 2016, COPD caused about 3.2 million deaths worldwide, in which low and middle-income countries (LMIC) accounted for more than 90% of those deaths [2]. The deaths related to CRDs in these LMIC are commonly due to lack of proper management [1,4,5]. In sub-Saharan Africa (SSA) including Tanzania, CRDs, in particular, COPD and Asthma have been a silent killer of many people [6,7].

The established commonest cause of CRDs is tobacco smoking, and indoor air pollution [2]. However, in SSA the burden and risks of CRDs are high greatly fueled by indoor air pollution from the high-level use of biomass fuel, HIV/AIDS pandemic, Pulmonary Tuberculosis (PTB) and pneumonia [7,8]. For instance, the proportion of households using biomass fuel in this region ranges from 86–99% and 26–96% in rural and urban areas respectively [9]. In Tanzanian, more than 90% of energy sources for cooking, heating, and lighting comes from solid biomass fuels such as firewood and charcoal [9]. Tobacco smoking prevalence is 14% for men and 0.6% in females [10], however, the COPD burden is also high among the female population due to indoor air pollution during cooking. The previous study conducted in rural Tanzania found that the prevalence of COPD was 17.5% (21.7% in males and 12.9%), and the associated causal risks were exposure to biomass fuel, pulmonary tuberculosis, and tobacco smoking [11]. On the other hand, the prevalence of self-reported Asthma among secondary school student in urban and rural Tanzania was found to be 17.6% and 6.4% respectively [12]. Furthermore, CRDs in particular COPD is potentially associated with extra-pulmonary manifestation including cardiovascular disease (CVD) hence patients are more likely to die from acute cardiovascular events than the primary lung pathology [13]. The extra-pulmonary manifestations of COPD also have been reported in Tanzania among patients with COPD in which 15.4% and 24% of study participants had CVDs and albuminuria (a marker of vascular endothelial dysfunction) respectively [14].

Generally, management of patients with CRDs is based on diagnosis confirmation and proper management of the symptoms to prevent acute exacerbation [15]. Despite the spirometry is the Gold standard for diagnosis of CRDs, a peak flow meter can be used to diagnose bronchial asthma in repeated measurement. However, it should not be used as a substitute for the spirometry [15]. In management or control of the symptomatic CRDs may include the use of inhaled bronchodilators (e.g. salbutamol), and corticosteroids like beclomethasone and budesonide [15,16]. These basic medicines are of very important for life serving and as controller of disease symptoms. However, previous studies showed that there is a scarcity of diagnostic equipment and basic medicines for CRDs at many health facilities in SSA including Tanzania [7]. Traditionally, the Tanzanian health system is designed to provide Reproductive Health services and manage communicable diseases which are more prevalent than non-communicable diseases (NCD). Despite the increase in NCD cases that create a double burden situation within the country, the budget and resources allocation is still insufficient for management of NCD such as CRDs [17]. Recent studies in Tanzania have shown that the primary health care facilities are not adequately equipped to respond to the increasing burden of hypertension and diabetes mellitus [18,19]. However, it is not known whether or not the healthcare facilities are adequately equipped and ready to provide management for CRDs.

Therefore, this study sought to explore the availability of services and factors associated with the readiness of health facilities to manage CRDs in Tanzania. This will help to inform the policymakers the area which requires necessary improvement given the high burden of CRDs and associated risk factors.

## Materials and methods

### Data source

The current study based on the secondary data analysis of the 2014–2015 Tanzania Service Provision Assessment (TSPA) Survey. The original TSPA survey was conducted by the Tanzania Ministry of Health and Social Welfare (MoHSW). Technical support for the survey was provided by ICF International under the DHS Program. The United States Agency for International Development (USAID/Tanzania) provided financial support. The TSPA survey was designed to gather important information on the availability and readiness of the basic health care services at Tanzania health facilities. The aim was to assess the presence and function of components essential for quality services delivery in the following areas; NCD, Family Planning (FP), Antenatal care (ANC), Child Health, Maternal and Newborn Care, Sexually Transmitted Infections, Malaria, Tuberculosis, and HIV/AIDS [20].

### Sampling technique and sample size

The TSPA survey was a sample survey of all formal-sector health facilities in Tanzania. A master list of health facilities that consisted of 7,102 verified (active) health facilities in Tanzania was obtained from the MoHSW in the Tanzania Mainland and the Ministry of Health (MOH) in Zanzibar. The list included hospitals, health centers, dispensaries, and clinics. These facilities were managed by the government, private-for-profit, parastatal, and faith-based entities. This survey used four main types of data collection tools; Facility inventory questionnaire, Health Provider Interview questionnaire, Observation Protocols ANC, FP, and services for sick children, and Exit Interview questionnaires for ANC and FP clients and for caretakers of sick children whose consultations were observed. The TSPA survey designers randomly selected a total of 1200 facilities for inclusion in the survey. This sample was designed to provide nationally-representative results according to the type of facility, managing authority, and regions for both Tanzania Mainland and Zanzibar [20].

**Fig 1:**
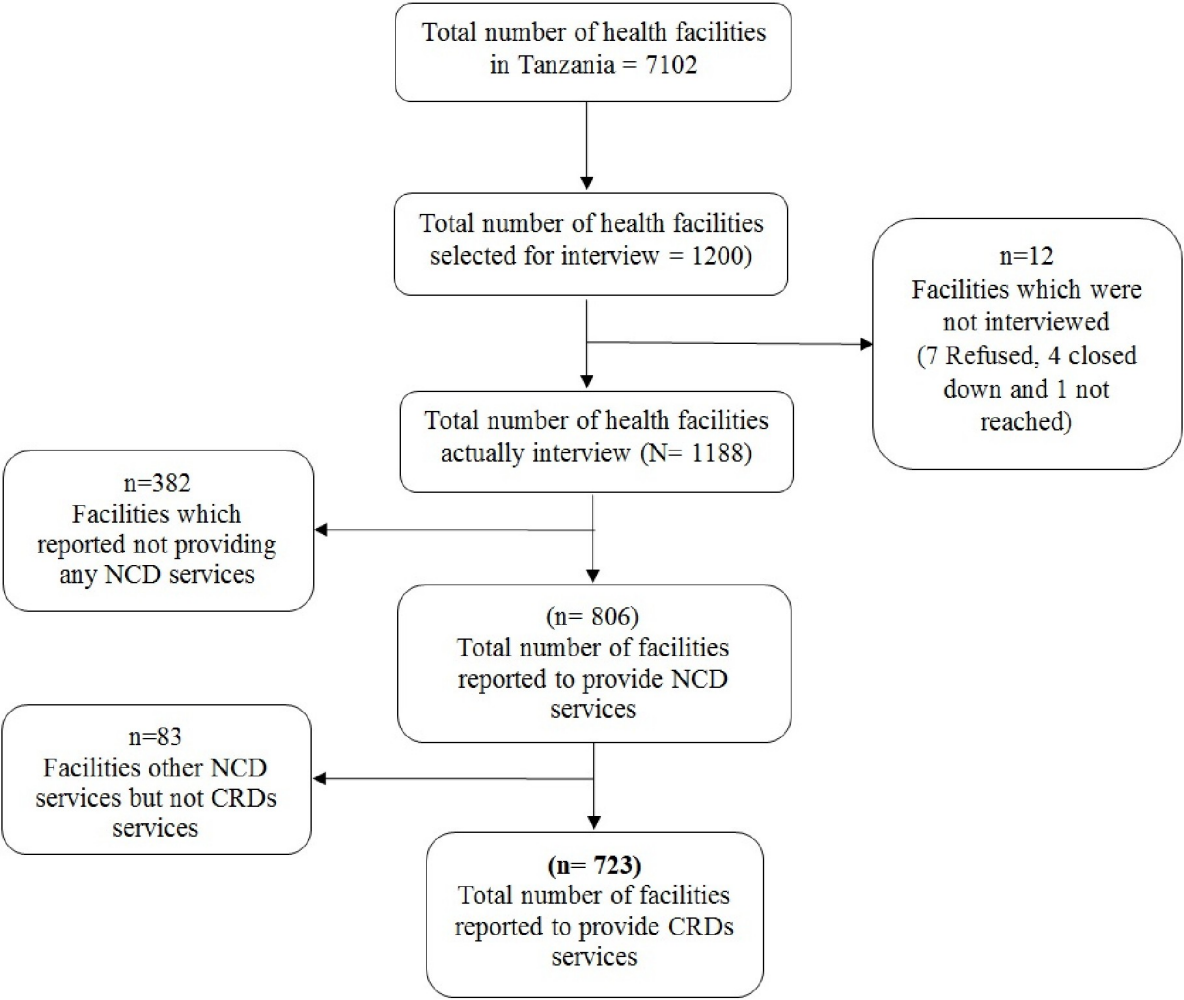
Selection of sampling units (health facilities) included in this analysis.

For the purpose of the current study, we analyzed data from Facility inventory file, therefore, the unit of analysis remained at the facility level. The main inclusion criteria were; the health facility must have agreed to participate in the survey, being opened during the day of the interview, and providing CRDs services. Out of 1200 facilities, the following were excluded because of not meeting the inclusion criteria; seven refused to participate, four were closed on the day of the survey, 465 were not providing CRDs services. Therefore, only 723 health facilities were included in the current analysis, Fig 1.

### Definition of research terms

#### Readiness

In this study is defined as "the capacity of the health facility to provide outpatient care for chronic respiratory diseases". The facility was considered to have “high readiness” if they scored at least a half (≥50%) of the indicators in each of the three domains suggested by World Health Organization-Service Availability and Readiness Assessment (WHO-SARA) reference manual. The domains were; staff training and guideline, equipment (peak flowmeter, spacer devices, stethoscopes, and oxygen cylinders), and basic medicines (injectable epinephrine, hydrocortisone tablets, prednisolone tablets, salbutamol, and beclomethasone inhalers). Otherwise, the facility was considered to have “low readiness" if they scored less than half (<50%) of the aforementioned indicators in each of the three domains.

#### Chronic respiratory diseases services

In this study is defined as "the ability of the facility to provide diagnosis and/or management of chronic respiratory diseases".

### Measurement of variables

#### Outcome variable

The outcome variable in this study was "readiness" of the health facilities. The readiness variable was rated as an index categorized into three domains as suggested by the WHO-SARA reference manual [21]. The first domain was staff and guidelines which had two indicators; the presence of a guideline for the management of CRDs and at least one staff who received a refresher training to manage CRDs. The facilities with guidelines were categorized as “Yes” while those without such guidelines were categorized as “No.” similarly, facilities with at least one staff member that had received refresher training in CRDs within 24 months were categorized as “Yes,” otherwise were categorized as “No”. The second domain was equipment which had four indicators; the peak flow meter, spacer devices, stethoscopes, and oxygen cylinders. Each equipment indicator was categorized as “Yes” for facilities that had reported the presence of such an indicator and "No" for facilities reported the absence of such an indicator. The third domain was a basic medicine which had five indicators: the availability injectable epinephrine, hydrocortisone and prednisolone tablets, salbutamol, and beclomethasone inhalers. Each medicine was categorized as “Yes” for facilities that had reported the availability of that indicator and "No” for facilities that had reported the absence of such an indicator. The responses were aggregated into an index score to calculate a composite score as per the WHO-SARA reference manual [21]. The index score was calculated by adding the presence of each indicator, with equal weight given to each of the domains and each of the indicators within the domains. Since the target was 100%, each domain accounted for 33.3% (100%/3) of the index. The percent for each indicator within the domain was equal to 33.3% divided by the number of indicators within that domain. Then, the facility that scores at least half (equivalent to the median value of 16.7% and above) in each domain and adding up to the overall of 50% or more were considered to have “high readiness” for the outpatient management of CRDs while those with less than 50% were considered to have “low readiness.” The cut-off point used in this study was also used elsewhere to dichotomize the outcome variable [19,22–24].

#### Explanatory variables

The facility type was categorized as “hospital,” “health center,” and “clinic/dispensary”; location was categorized as “mainland” and “Zanzibar”; residence was categorized as “urban” and “rural”; managing authority was categorized as “public” and “private”; routine management meetings were categorized as “performed” for facilities conducting routine management meetings and “not performed” for facilities that reported not having routine management meetings; external supervision was categorized as “received” for facilities that reported that had received external supervision and “not received” for facilities that reported had not received external supervision; external source of revenue was categorized as “none,” “government” and “none government”; CRDs guideline was categorized as "available" for facilities that had reported the availability of any type of NCDs guideline otherwise were categorized as “not available”; user fees was categorized as “fixed for all services” for facilities that reported to have a fixed cost for all types of services the patients would receive otherwise were categorized as “separate”; training about NCD was categorized as “available” for facility that reported to have at least one staff member who had received training in NCDs, otherwise were categorized as “not available”.

### Data processing and analysis

Data were analyzed using Stata 14 (StataCorp, College Station, TX). For all analyses, the “svy” set command in Stata was employed to adjust for the complex sampling design used in the TSPA survey. All estimates were weighted to correct for non-responses and disproportionate sampling. In the descriptive analysis, the categorical variables were summarized using frequency and proportions and then presented either in tables or graphs when appropriate. An unadjusted logistic regression model was fitted to assess whether the is an association between the outcome variable “readiness” and the explanatory (health facility) variables; location, residence, facility type, managing authority, management meeting, external supervision, an external source of revenue, type of user fees and acceptability of health insurance. Thereafter, all explanatory variables that showed association with a *P* value *<* 0.2 were fitted into the multivariable logistic regression model using the stepwise (backward elimination) method to test for the association of each variable with the outcome variable. In addition, we disaggregated the outcome variable into three predetermined domains (staff and guidelines, diagnostic and equipment, and essential medicines) and then fitted three multivariable logistic regression models to identify separate factors for each domain. However, in this study, we presented and discussed only the results of composite outcome variable “readiness.” The separate models are summarized in S1 Table. A *P*-value of less than 0.05 was considered statistically significant. The generalized variance inflation factor (VIF) was performed to test for the multicollinearity, which usually should not exceed 4.0. In the current analysis, no any of the variable included in the model presented with VIF>2.0, thus suggesting no any doubts for multicollinearity. The resulting final model included all the variables that determine the "readiness" of the facility to provide CRDs services. The *P*-value and the 95% confidence interval (CI) for the odds ratio (OR) were used to ascertain the significance of the associations.

### Ethical considerations

This study was based on an analysis of existing public domain survey datasets that are freely available online with all identifier information detached. The original TSPA survey was approved by Tanzania's National Institute for Medical Research (NIMR), the Zanzibar Medical Ethics and Research Committee (ZAMREC) and the Institutional Review Board of ICF International in the USA. The informed consent was requested and obtained from the manager, the person-in-charge of the facility, or the most senior health worker responsible for client services who was present at the facility. All relevant aspects of the study, including its aim and interview procedures were explained clearly to the respondents before interviews. Those respondents agreed their facilities to participate in the study, provided a signed written informed consent.

## Results

### Distribution characteristics of health facilities

Table 1 represents the proportion distribution of the health facilities reported to provide management for CRDs according to background characteristics. Of the 723 health facilities, 68.6% were public managed, 93.7% belonged to primary health care (clinic, dispensary, and health center), 70.9% were located to urban, and 81.2% had routine management meetings conducted. On the other hand, only 26.0% and 13.0% of health facilities had guidelines and staff trained staff for NCDs respectively.

**Table 1:** Percent distribution of surveyed facilities according to background characteristics, Tanzania SPA 2014–15 (n = 723).

Variable	n (%) (weighted)
**Facility location**	
Mainland	681 (94.2%)
Zanzibar	42 (5.8%)
**Managing authority**	
Public	496 (68.6%)
Private	227 (31.4%)
**Facility type**	
Clinic & dispensary	565 (78.1%)
Health center	113 (15.6%)
Hospital	45 (6.3%)
**Facility residence**	
Urban	210 (70.9%)
Rural	513 (29.1%)
**Routine management meetings**	
Not performed	136 (18.8%)
Performed	587 (81.2%)
**External supervision**	
Not received	16 (2.2%)
Received	707 (97.8%)
**External source of revenue**	
Government	391 (54.1%)
Other than government	258 (35.7%)
None	74 (10.2%)
**User fees**	
Fixed for all services	429 (59.3%)
Separate for each service	294 (40.7%)
**Health insurance**	
Not accepted	422 (58.4%)
Accepted	301 (41.6%)
**Guidelines for NCD**	
Not available	535 (74.0%)
Available	188 (26.0%)
**Staff trained about NCD**	
Not available	629 (87.0%)
Available	94 (13.0%)

### Availability of important components for management of CRDs

**Fig 2:**
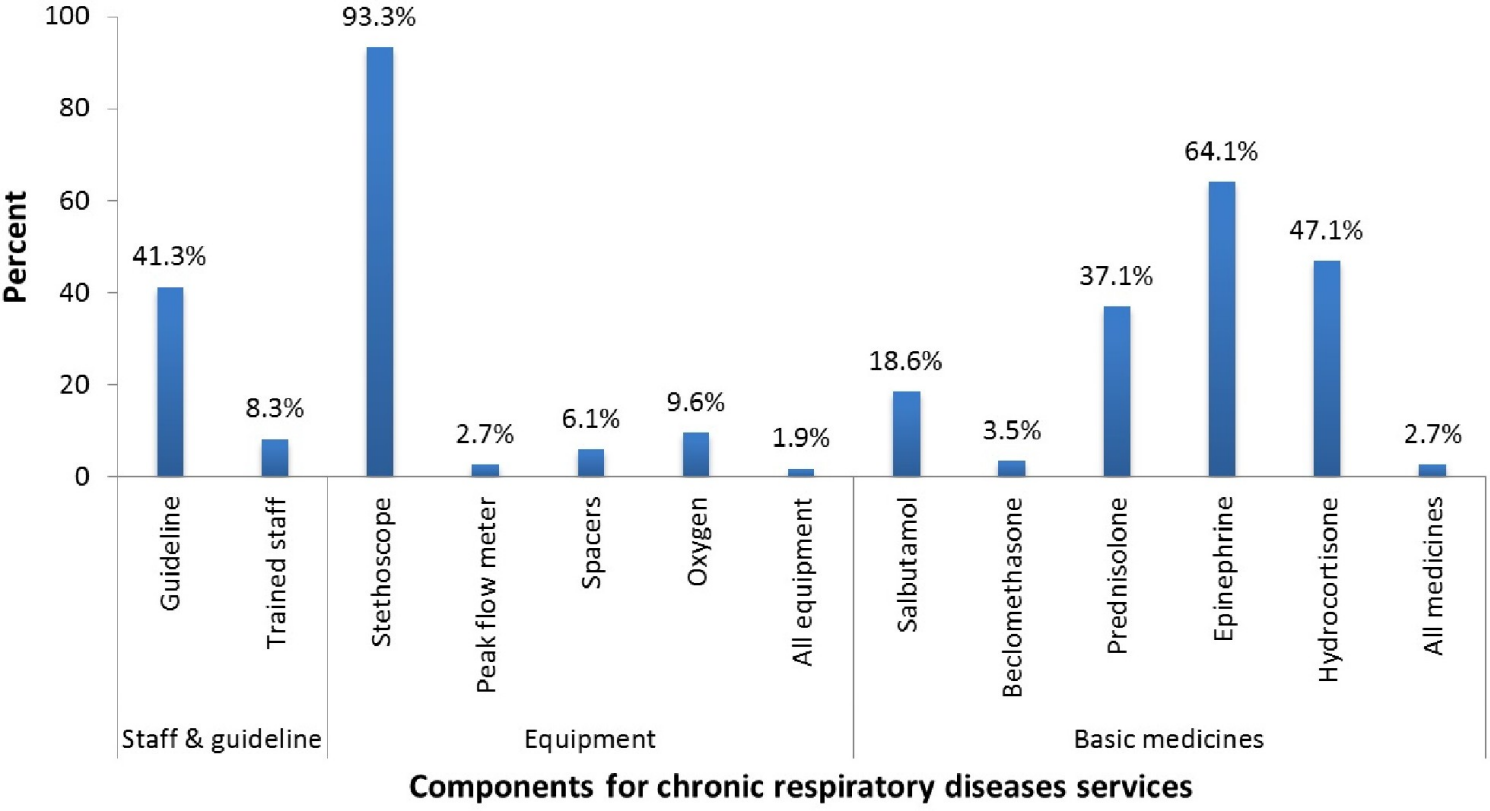
Availability of basic components that support the provision of CRDs (N = 723).

Fig 2 represents the availability of important components for managing CRDs at health facilities. Of the 723 health facilities reported to provide management for CRDs, 41.3% had guidelines, 8.3% at least one trained staff, 1.9% equipment, and 2.7% basic medicines for the management of CRDs. If an individual component is considered separately; for basic medicines component, 64.1% and 47.1% of health facilities had injectable epinephrine and hydrocortisone respectively while only 18.6% of the health facilities had inhaled salbutamol available. Regarding equipment component; 93.3% of health facilities had stethoscope while less than one-tenth of health facilities had each of the following available oxygen, peak flow meter, and spacers.

### Facility readiness to provide management for CRDs

**Fig 3:**
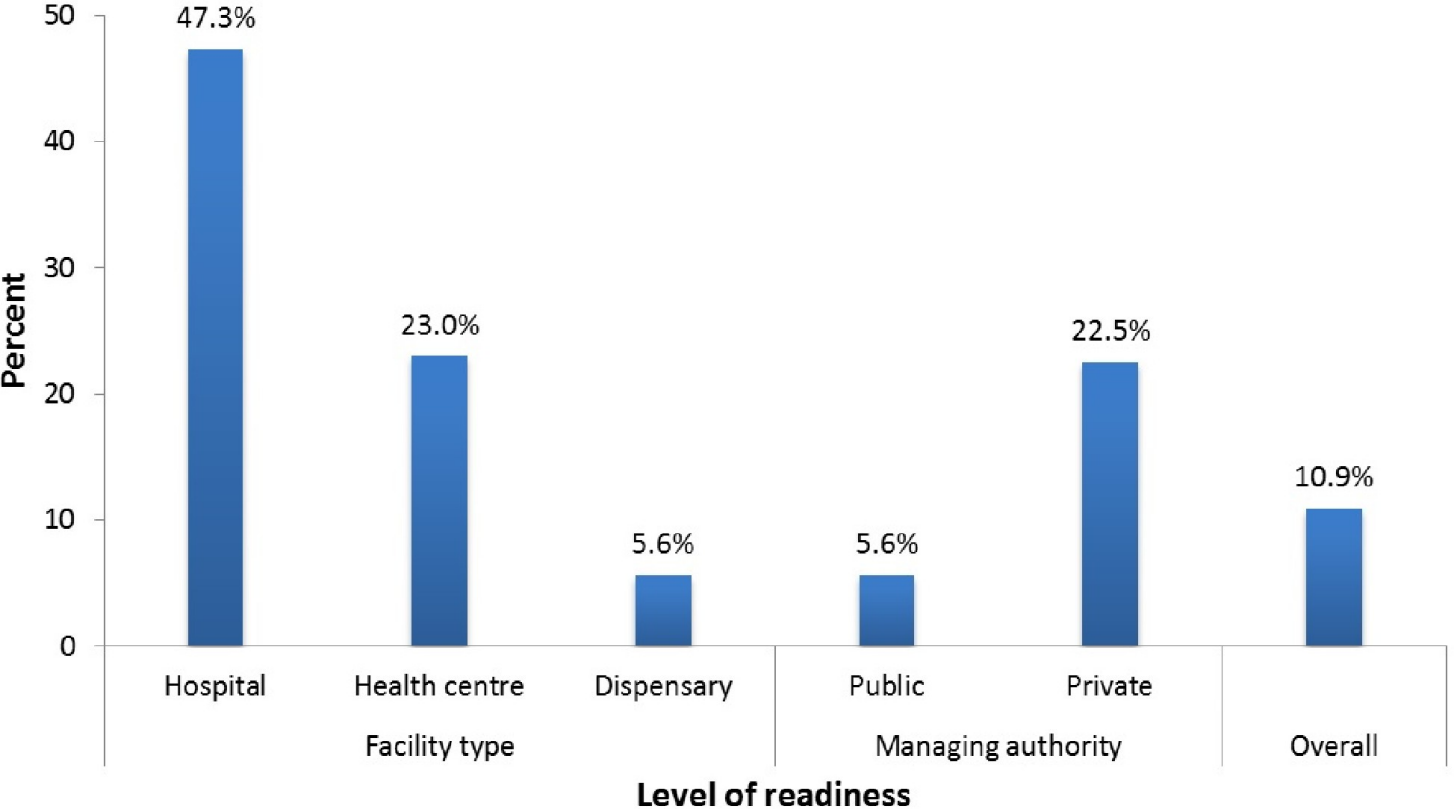
Percentage distribution of facility readiness for management of CRDs according to facility type and managing authority.

Fig 3 represents the level of readiness of facilities to provide management for CRDs by facility type and managing authority. In an overall, only 10.9% of the facilities had a high readiness to provide management for CRDs. Despite this low proportion, after stratifying the readiness according to facility type, hospitals performed better than other facilities to provide management for CRDs. Moreover, based on managing authority the majority of public facilities had the low readiness to provide management for CRDs.

### Factors associated with readiness to provide management of CRDs

Table 2 represents the logistic regression of the factors associated with facility readiness to provide management for CRDs. In adjusted model the following factors were significantly associated with likelihood of health facility having high readiness to provide management for CRDs; private owned facilities [AOR = 3.3, 95% CI; 1.5–7.5], facility level (health centres [OR = 5.0, 95% CI; 2.4–10.7], hospitals [OR = 11.6, 95% CI; 5.0–27.2]), and having routine management meetings [AOR = 3.3, 95% CI; 1.4–7.8].

**Table 2:** Factors associated with facility readiness for outpatient management of CRDs, adjusted for confounding variables, Tanzania SPA 2014–15 (*n* = 723).

Variable	COR [95% CI]	AOR [95% CI]
**Facility location** (ref: Mainland)		
Zanzibar	1.4 [0.7–2.6]^#^	
**Managing authority** (ref: Public)		
Private	4.9 [2.9–8.5]	3.3 [1.5–7.5]*
**Facility type** (ref: Clinic & dispensary)		
Health center	5.1 [2.6–9.8]	5.0 [2.4–10.7] **
Hospital	15.2 [7.7–30.3]	11.6 [5.0–27.2]**
**Facility residence** (ref: Rural)		
Urban	4.1 [2.3–7.3]	1.7 [0.9–3.1]
**Routine management meetings** (ref: Not performed)		
Performed	6.2 [2.9–13.4]	3.3 [1.4–7.8]*
**External supervision** (ref: Not received)		
Received	2.1 [0.6–7.2]^#^	
**External source of revenue** (ref: Government)		
Other than government	1.1 [0.6–2.1]	0.9 [0.4–2.1]
None	3.4 [1.5–7.9]	2.0 [0.6–6.3]
**User fees (ref:** Fixed for all services)		
Separate for each service	3.3 [1.9–5.8]	1.3 [0.6–2.8]
**Health insurance** (ref: Not accepted)		
Accepted	0.7 [0.5–1.2]^#^	

Note

* = *P*-value < 0.05

** = *P*-value < 0.001

^#^ = *P*-value ≥ 0.2 therefore, the variables were not included in the multivariable model

## Discussion

In this article, we examined the availability of services and level of health facilities readiness to provide management for CRDs and its associated factors in Tanzania. Our study revealed the unsatisfactory proportion of Tanzanian health facilities with a high level of readiness to provide management for CRDs. In addition, it found that privately owned facilities, high-level facilities (hospitals and health centers), and performance of routine management meetings were significantly associated with a high readiness to provide management for CRDs.

The WHO strategy for prevention and control of CRDs recommends provision of refresher training to healthcare providers and ensure availability, accessibility, and affordability of guideline, diagnostics equipment, and basic medicines for the management of CRDs [1,25,26]. However, the current study found the low proportion of health facilities with the availability of guidelines, trained staff, equipment, and basic medicines necessary for management of CRDs. These findings are in agreement with those reported in previous studies conducted in other SSA countries [27–30]. Furthermore, the current study found that the overall proportion of health facilities readiness to provide management for CRDs was very low. Given the existing epidemiological risks and burden of CRDs in Tanzania context, there is an urgent need to address the availability of important components in all health facilities across the country regardless of facility type and managing authority. Otherwise, this may lead to under-recognition, under-diagnosis, and under-treatment of CRDs hence fueling the epidemic of NCD in Tanzania.

The current study found that private owned facilities and high-level facilities (health centers and hospitals) were more likely to have a high readiness to provide management for CRDs compared to publicly owned facilities and lower-level facilities (dispensaries) respectively. The observed finding might be explained by the evidence that private owned health facilities in the LMIC setting have sustainable healthcare provision in terms of responsibility, accountability, and efficiency than publicly owned health facilities [31,32]. Also, might be due to the fact that publicly owned facilities have a long process (involving multiple levels of authorization) in ordering and procuring of equipment and basic medicines for facility consumption [33,34]. This long process may result in the low availability of equipment and medicine, therefore, may explain the high proportion of publicly owned facilities to have low readiness for management of CRDs.

Moreover, the current study found that facilities that conducted routine management meetings were significantly more likely to have a high readiness to provide management for CRDs compared to their counterpart. The performance of routine management meetings may have a great influence in clinical decision making especially when it is reflected in complex cases scenario of CRDs. This may motivate the health facility authority to make available of necessary components for managing CRDs. The current study finding is comparable to the previous study in Tanzania which found that performance of routine management meetings was significantly associated with health facility being prepared to provide management for hypertension [19]. Therefore, the current findings suggest that routine management meetings may an independent factor that can influence the readiness of health facilities to provide management for CRDs.

In addition, facility residence (rural or urban) was found to be the not significant associated with the high readiness of the health facility to provide management for CRDs. In Tanzania, more than 70% of the population lives in a rural setting which is served mostly by public health facilities [10]. Moreover, the risk of CRDs from indoor air pollution due to the use of solid biomass fuel in a rural setting is high [9]. Hence this study highlights the urgent need to improve important infrastructure necessary for health facilities readiness to provide management for CRDs. Therefore, the government should strive to improve the availability of components for management of CRDs in particular to the public health facilities which serve the majority of the population.

The significance of the current study relies on the unique epidemiology of CRDs in Tanzania [11,12,14] and other countries in SSA [7,8,35]. Besides tobacco smoking which is prevalent among male population [36,37] the other important potential risks for CRDs have been indoor air pollution from a high level of biomass fuel use [8–10], a respiratory infectious disease in particular pulmonary tuberculosis (PTB) and pneumonia. Post pulmonary tuberculosis treatment sequelae include bronchiectasis and fibro-thorax which are the components of CRDs [26,38,39]. The association between CRDs and biomass fuels and tuberculosis have been reported in Tanzania [11] and other African countries [7,40]. Therefore, the epidemiology of CRDs in Tanzania is of its own uniqueness in the shadow of infectious diseases and solid biomass fuel use. Given this circumstance, it is of paramount importance to have service available for management of CRDs in all health facilities in the Tanzania context.

The current study has the following strengths; it is the first study to assess the readiness of the health facilities in Tanzania to provide management for CRDS. The study used a nationally representative sample in which our findings reflect the actual situation of the health facilities regarding management of CRDs. However, the cross-sectional nature of the study limits its utility to explain the relative proportion and temporal relation of all the causative factors to the burden of CRDs. Therefore, the results should be interpreted with caution. In addition, the current study did not assess the availability of other important components for management of CRDs such as diagnostic spirometry and nebulizer machines; this might have changed the obtained results.

## Conclusion

Majority of Tanzanian health facilities have low readiness to provide management for chronic respiratory diseases. Privately owned facilities, higher-level facilities (hospitals and health centers), and performance of routine management meetings were significantly associated with high readiness to provide management for CRDs. There is a need for the Tanzanian government to increase the availability of diagnostic equipment, medication, and to provide refresher training specifically in the lower-level and public health facilities for better management of CRDs and other non-communicable diseases.

## Supporting information

S1 Table Disaggregated analysis according to three domains to identify factors associated with facility readiness for outpatient management of CRDs, Tanzania SPA 2014–15 (*n* = 723). (DOCX)
