# Supplementary material for: Are Tanzanian health facilities ready to provide management of chronic respiratory diseases? An analysis of national survey for policy implications
Source: PLoS One. 2019 Jan 7;14(1):e0210350. doi: 10.1371/journal.pone.0210350 (PMC6322729; doi:10.1371/journal.pone.0210350)
Supplement: S1 Table — (DOCX) [file pone.0210350.s001.docx]

**S1 Table Disaggregated analysis according to three domains to identify factors associated with facility** readiness **for outpatient management of CRDs, Tanzania SPA 2014-15 (*n* = 723)**

|  | **Staff and guidelines** | **Equipment** | **Medicines** |
| --- | --- | --- | --- |
| **Variable** | **AOR [95% CI]** | **AOR [95% CI]** | **AOR [95% CI]** |
| **Facility location** (ref: Mainland) |  |  |  |
| Zanzibar | 1.2 [0.5 - 2.9] | 2.8 [1.0 - 7.9]* | 3.1 [1.7 - 5.9]** |
| **Managing authority** (ref: Public) |  |  |  |
| Private | 0.5 [0.2 - 1.3] | 5.1 [2.7 - 9.5]** | 4.8 [2.2 - 10.5]** |
| **Facility type** (ref: Clinic & dispensary) |  |  |  |
| Health center | 6.4 [2.3 - 17.6]** | 6.9 [2.1 - 23.0]* | 2.1 [1.2 - 3.8]* |
| Hospital | 3.4 [0.9 - 13.3] | 10.6 [3.3 - 33.3]** | 7.2 [3.6 - 14.6]** |
| **Routine management meetings** (ref: Not performed) |  |  |  |
| Performed | 1.0 [0.4 - 2.7] | 28.6 [3.4 - 43.2]* | 2.9 [1.2 - 7.2]* |
| **External source of revenue** (ref: Government) |  |  |  |
| Other than government | 0.7 [0.4 - 1.4] | 2.8 [1.4 - 5.9]* | 0.9 [0.5 - 1.9] |
| None | 0.6 [0.1 - 2.6] | 2.9 [0.7 - 11.2] | 1.4 [0.5 - 3.6] |
| **User fees (ref:** Fixed for all services) |  |  |  |
| Separate for each service | 3.4 [1.9 - 6.3]** | 2.2 [1.0 - 5.0] | 1.4 [0.7 - 2.8] |

**Note:** *** = *P*-value < 0.05, ** = *P*-value < 0.001**
